# Host-Species Variation and Environment Influence Endophyte Symbiosis and Mycotoxin Levels in Chinese *Oxytropis* Species

**DOI:** 10.3390/toxins14030181

**Published:** 2022-02-28

**Authors:** Chenchen Guo, Li Zhang, Qianqian Zhao, Manfred Beckmann, Helen Phillips, Huizhen Meng, Chonghui Mo, Luis A. J. Mur, Wei He

**Affiliations:** 1 Key Laboratory of Resource Biology and Biotechnology in Western China, Ministry of Education, College of Life Sciences, Northwest University, Xi’an 710069, China; guochenchen@stumail.nwu.edu.cn; 2 Provincial Key Laboratory of Biotechnology of Shaanxi Province, College of Life Sciences, Northwest University, Xi’an 710069, China; xdzhangli@stumail.nwu.edu.cn (L.Z.); menghuizhen@stumail.nwu.edu.cn (H.M.); 3 Peiwen School of Peking University, Jinzhong 030619, China; zhaoqiansx@163.com; 4 Institute of Biology, Environmental and Rural Science, Aberystwyth University, Aberystwyth SY23 3FL, UK; meb@aber.ac.uk (M.B.); hcp5@aber.ac.uk (H.P.); 5 Agriculture and Animal Husbandry College, Qinghai University, Xining 810016, China; mchqwhmhg@qhu.edu.cn; 6 Key Laboratory of Grassland Resources of Ministry of Education, College of Grassland, Resources and Environment, Inner Mongolia Agricultural University, Hohhot 010018, China

**Keywords:** *Oxytropis* spp., genetic diversity, genetic structure, endophyte, *Alternaria* *oxytropis*, swainsonine

## Abstract

*Oxytropis* plants are widely distributed in the grasslands in northern China. Some *Oxytropis* species have been reported to contain the mycotoxin swainsonine, an alkaloid which causes poisoning in livestock, referred to as locoism. Previous studies showed that endophytic fungi (*Alternaria* *oxytropis*) symbiotically associate with these *Oxytropis* species to produce swainsonine. However, the influence of variation within the *Oxytropis* genus on the fixation or loss of symbiosis and toxicity is poorly understood, as is the influence of environmental factors. Here we used a collection of 17 common *Oxytropis* species sampled in northern China to assess genetic diversity using genotyping by sequencing which was compared with the levels of the endophyte and swainsonine. Results showed that nine *Oxytropis* species have detectable *A.* *oxytropis* colonisation, and seven *Oxytropis* species contain sufficient swainsonine to be considered poisonous, whereas the rest may be non-toxic. Species variation rather than the genetic lineage was associated with the fixation or loss of endophyte and swainsonine production, which appears to have resulted from genetic drift. Genotype × Environment (G × E) effects were also found to influence endophyte and swainsonine levels amongst species of the *Oxytropis* genus. Our study will provide a better understanding about the evolutionary basis of *A.* *oxytropis* symbiosis and swainsonine biosynthesis in locoweeds.

## 1. Introduction

The symbiosis between plants and endophytic fungi is common in nature [1,2,3], and the fungi have beneficial, neutral or detrimental effects to the host plants. Some hosts exhibit a preference for certain endophytic fungal interactions [4]. The diversity and distribution of endophytes across biomes and their effects on plant phylogeny and host tissues have attracted considerable attention [5]. *Oxytropis* spp. (Fabaceae) are important perennial herbs that are native to temperate to frigid zones in Europe, Asia and North America [6,7]. Some *Oxytropis* species form a symbiotic interaction with the endophytic fungi *Alternaria* (sect. *Undifilum*) *oxytropis*, to synthesise a mycotoxin, swainsonine [8,9,10]. Swainsonine, an indolizidine alkaloid, has the inhibitory activity of α-mannosidase [11,12] and can cause poisoning of livestock (mainly sheep and horses). Poisonous *Oxytropis* species, together with the poisonous *Astragalus* species, are known as “locoweeds”, and pose a serious threat to animal husbandry in China and the U.S. [6,13,14,15]. When combined with overstocking, poisonous *Oxytropis* species can become dominant in the plant community, as observed in many grasslands in China [16]. Therefore, understanding what can influence mycotoxin production in the *Oxytropis*–*A. oxytropis* symbiont is of considerable importance, not only to reduce the risk of livestock poisoning, but also to improve our knowledge of key aspects of host–endophyte symbiosis.

Swainsonine producing strains of *A.* sect. *Undifilum* are considered to form a commensal association with the two related genera of *Astragalus* and *Oxytropis* [17]. The levels of swainsonine vary in different plant species, tissues and at different phenological stages [18]. Another important variable influencing swainsonine concentrations in the plant is the chemotype of *A. oxytropis*, with high- and low-swainsonine-producing variants being identified in *O. sericea* [19]. This may reflect genetic variation in the swainsonine-producing gene cluster. Such a gene cluster has been identified in *A.* sect. *Undifilum*, comprising *SwnR*, *SwnN*, *SwnH1*, *SwnH2* and *SwnK* [20]. Swainsonine levels are also related to the amount of endophyte in a host population [21,22,23,24,25]. A recent study showed that different strains of *A. fulva* (also belonging to *A*. sect. *Undifilum*) may exist in the same host, but swainsonine concentration is largely determined by the amounts of the dominant chemotype [26].

Our previous study showed that *O. ochrocephala*, the most common toxic *Oxytropis* species in China, could be differentiated into two large genetically distinct groups, within which there were considerable variations in endophyte infection patterns and swainsonine content [27]. Different growth conditions also had an impact on the production of swainsonine, once the symbiosis was fixed in the host population. As such, host genetics and environments (G × E) together could have shaped the symbiosis and mycotoxin biosynthesis. However, the distribution of *A. oxytropis* in some of the other *Oxytropis* species remains cryptic. Swainsonine production in different *Oxytropis* species has been linked to the same conspecies, designated as *A. oxytropis* [21,22,23,28]. Nevertheless, since *A. oxytropis* has been reported as strictly vertically transmitted [24], it is difficult to understand how the same endophyte is differently distributed between different locoweed species which are reproductively isolated. This may reflect the monophyletic maintenance or loss of endophytic symbiosis at divergence from a common *Oxytropis* ancestor. Alternatively, this could arise from independent variation occurring post-divergence. In this study, we aimed to characterise the genetic diversity of both swainsonine-positive and swainsonine-negative *Oxytropis* hosts. By determining the endophyte and swainsonine levels, we considered whether genetic lineage within the genus could be related to the colonisation of *A. oxytropis* and swainsonine biosynthesis. We also addressed the relationship between plant genetics, environment parameters, endophyte and swainsonine content. The results of our study suggested *A. oxytropis* symbiosis and swainsonine biosynthesis in *Oxytropis* may be influenced by G × E effects.

## 2. Results

### 2.1. Genetic Evolution of the Natural Population of Oxytropis *spp.*

Data obtained from 181 experimental samples of SLAF-seq (Specific-Locus Amplified Fragment Sequencing) were derived including 170 plant individuals from 17 *Oxytropis* species, with 10 randomly selected samples as replicates and *Medicago truncatula* as the outgroup. On average, Q30 was 97.13% and the GC content was 41.31%. The average sequencing depth was 19.46×, with these sequences revealed as 21,539,214 SLAFs. A total of 1,239,417,229 SNPs were identified.

The K2P model measured the genetic similarity matrix between the species. The genetic distance (GD) value of the *Oxytropis* populations of the 17 species was 0.0026~0.1032 (Table S1). The analysis revealed a large genetic distance between the populations, high overall genetic differentiation and a wide genetic basis. Among the species, *O. glabra* (GLA) and *O. myriophylla* (MY) were the most distant species, whereas *O. giraldii* (GI) and *O. melanocalyx* (MEL) were the closest. Nucleotide diversity (PI) was used to show the extent of genetic diversity amongst the 17 species. *O. aciphylla* had the largest PI (0.000114), and the PI of *O. glabra* was the smallest (0.000057) (Table S2).

**Figure 1:**
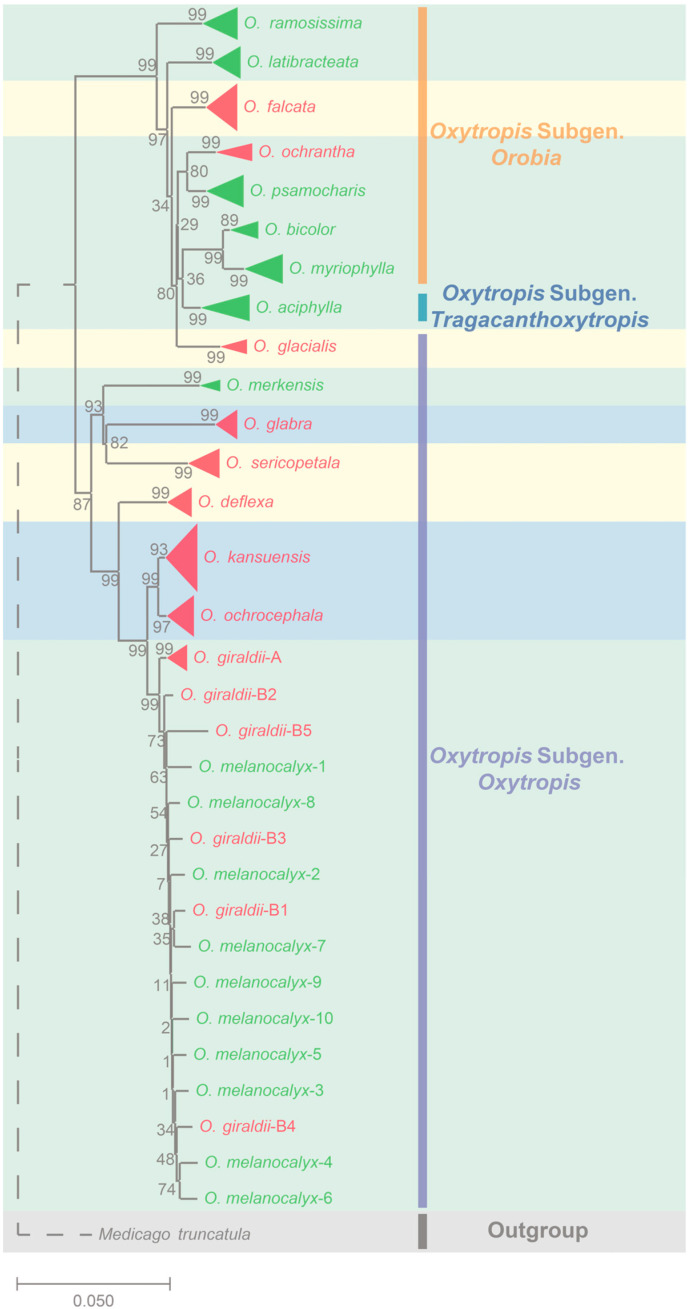
Neighbour-joining tree of the 181 individuals of the 17 *Oxytropis* species. *Medicago truncatula* was used as the outgroup. Samples clustered into a single clade and belonging to the same species are displayed by triangles. The size of each triangle represents the number of samples merged here, proportionally. The colour of the triangle and the label represents whether the corresponding sample contained endophyte *A. oxytropis*; red/green represent +/− symbiosis with *A. oxytropis*, respectively. Subgenus classification in traditional *Oxytropis* taxonomy is indicated by bars with different colours. Orange: *Oxytropis* subgen. *Orobia*; blue: *Oxytropis* subgen. *Tragacanthoxytropis*; purple: *Oxytropis* subgen *oxytropis*. The background colours represent different swainsonine content. Light yellow: swainsonine content > 0.1% (chemotype I); light green: swainsonine content < 0.01% (chemotype II); light blue: swainsonine content: 0.01–0.1%. Values next to nodes correspond to bootstrap values. The dotted line indicates that the substitution rate was not proportional.

Phylogenetic assessment indicated two different clusters among the 17 *Oxytropis* species (Figure 1). This generally agreed with the subgenera classification, where Clade I (top) included eight species belonging to the *Oxytropis* subgen. *Orobia*, the *Oxytropis* subgen. *Tragacanthoxytropis* and a species of the *Oxytropis* subgen. *Oxytropis*; and Clade II (bottom) included nine species but only in the *Oxytropis* subgen. *Oxytropis* (available at http://www.cfh.ac.cn/ (accessed on 30 November 2021)). Most of the plant individuals from the same species clustered together and formed their own branch, except *O. giraldii* (GI) and *O. melanocalyx* (MEL), which are morphologically similar and had the least genetic distance between them.

**Figure 2:**
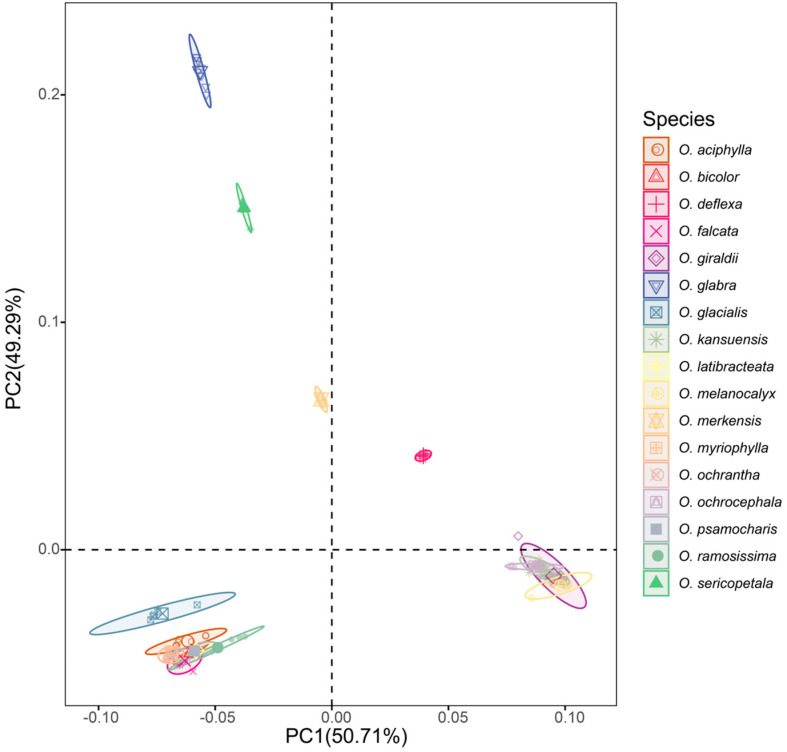
The principal component analysis (PCA) based on the entire SNP set for the 17 *Oxytropis* species.

The set of 1,239,417,229 SNPs (after removal of SNPs with MAF less than 0.05) were used for PCA analysis (Figure 2). Two principal components (PC) accounted for 49.29% and 50.71% of total variability, respectively. The 17 *Oxytropis* species could be arbitrarily divided into six different groups. From the top to the bottom they were as follows: (i–iv) the first four groups were composed exclusively of *O. glabra*, *O. sericopetala*, *O. merkensis* and *O. deflexa*, respectively; (v) the fifth plurispecific group comprised *O. giraldii*, *O. melanocalyx*, *O. kansuensis* and *O. ochrocephala*; and (vi) the sixth plurispecific group contained *O. myriophylla*, *O. bicolor*, *O. ramosissima*, *O. psamocharis*, *O. aciphylla*, *O. ochrantha*, *O. glacialis*, *O. falcata* and *O. latibracteata*, which is in accord with Clade I in the phylogenetic tree.

**Figure 3:**
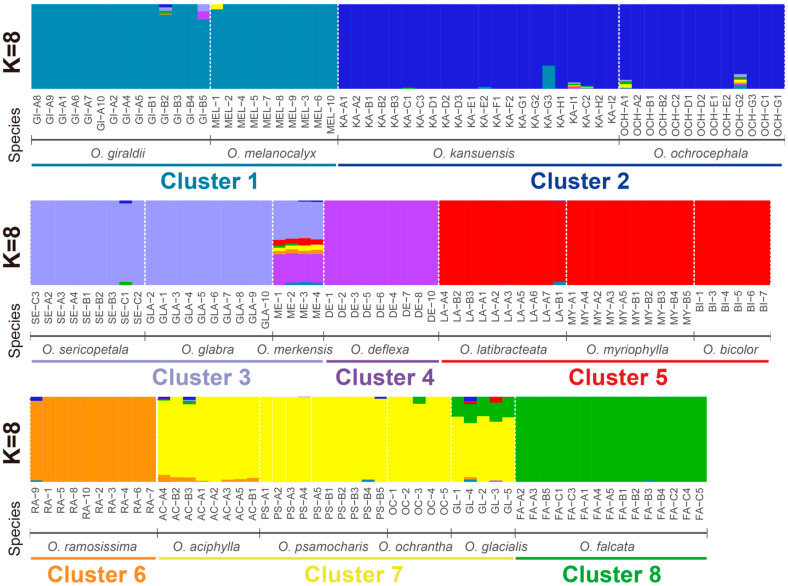
Population structure of *Oxytropis* estimated by ADMIXTURE. Each color represents one ancestral population (K = 8). Each individual is represented by a bar, and the length of each colored segment in the bar represents the proportion contributed from the ancestral population.

**Figure 4:**
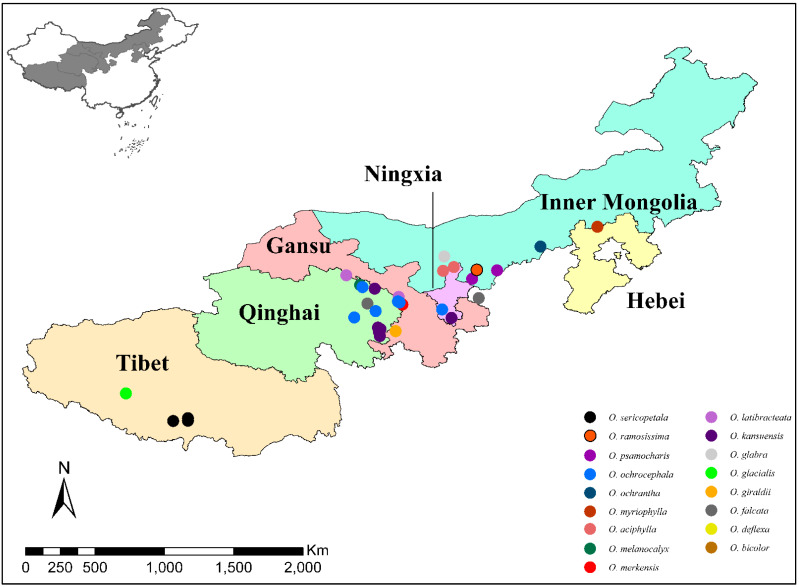
The geographical locations of *Oxytropis* plants sampled.

**Figure 5:**
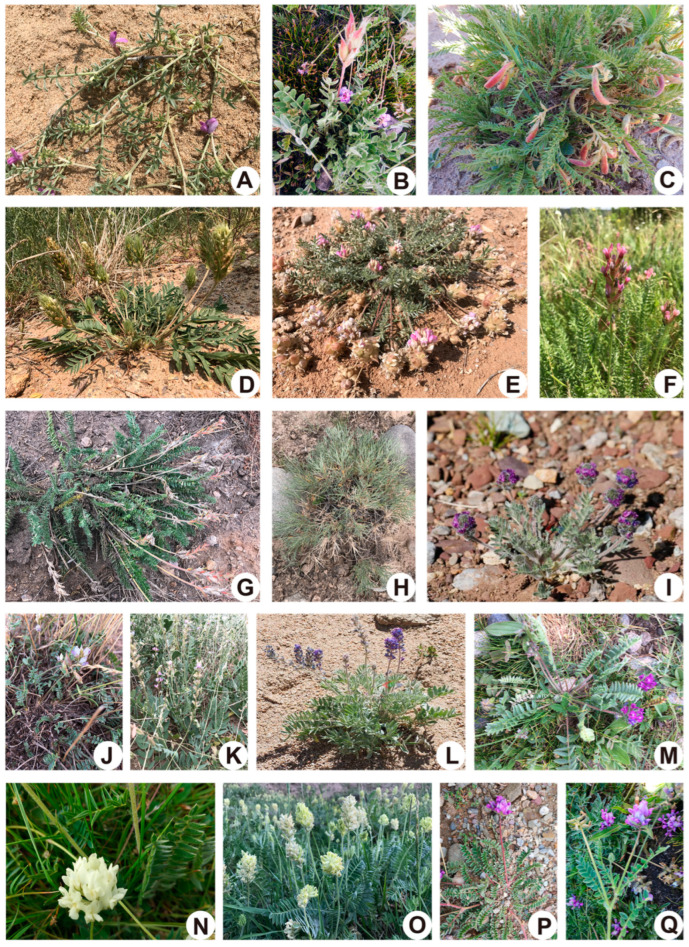
Morphological diversity of representative species of the *Oxytropis* genus. (**A**) *O. ramosissima* (**B**) O*. latibracteata* (**C**) *O. falcata* (**D**) *O. ochrantha* (**E**) *O. psamocharis* (**F**) *O. bicolor* (**G**) *O. myriophylla* (**H**) *O. aciphylla* (**I**) *O. glacialis* (**J**) O*. merkensis* (**K**) *O. glabra* (**L**) *O. sericopetala* (**M**) *O. deflexa* (**N**) *O. kansuensis* (**O**) *O. ochrocephala* (**P**) *O. giraldii* (**Q**) *O. melanocalyx*. Among those, *O. glacialis* and *O. sericopetala* were illustrated using the records from the Chinese Field Herbarium and the rest of the photos were taken during the sampling collection.

Admixture analysis was used to reveal the genetic structure of the samples. K = 8 was determined to be the most likely as indicated by CV errors (Figure S1) and we saw the admixture results of eight clusters (Figure 3, Figures S1 and S2). Cluster 1 contained both *O. giraldii* (GI) and *O. melanocalyx* samples. This agreed with the phylogenetic tree in which the two species cannot be resolved. Two individuals from GI-B, which were all collected from Qinghai Province (Figure 4, Table S3), showed genetic mixture with other clusters. Cluster 2 included *O. kansuensis* and *O. ochrocephala* composed of 35 samples from wide-ranging sampling sites (Qinghai, Gansu and Ningxia) (Figure 4, Table S3). The two species have similar morphological characteristics, such as flower colour and leaf morphology (Figure 5). Cluster 1 and 2 together represented the unresolved groups in PCA (group v). Clusters 3 and 4 contained the four well-separated species in PCA (group i–iv). *O. sericopetala*, *O. glabra* and *O. merkensis* formed Cluster 3, corresponding to their grouping in the phylogetetic tree, but in Cluster 4, *O. deflexa*, seemed to have introgressed into *O. merkensis*. This is also different from the evolutionary tree, in which *O. deflexa* shares an ancestor node with *O. giraldii*, *O. melanocalyx*, *O. kansuensis* and *O. ochrocephala* (Clusters 1 and 2). The admixture results of Clusters 5–8 distinguished the nine mixed samples in PCA (group vi) from each other. Samples of *O. latibracteata*, *O. myriophylla* and *O. bicolor* formed Cluster 5. Cluster 6 was formed by *O. ramosissima* only. Cluster 7 comprised *O. aciphylla*, *O. psamocharis*, *O. ochrantha* and *O. glacialis*, which showed considerable genetic admixture, especially in *O. glacialis* where *O. falcata* as Cluster 8 had introgressed. Compared with the phylogenetic tree, Clusters 1–4 relate to Clade I, and Clusters 5–8 relate to Clade II.

AMOVA analysis showed that 67.66% of the total genetic variation was between the 17 species, with 4.26% between sampled populations (from different geographical locations) within each species, and 28.09% between individuals within each of the 39 populations (Table 1). The fixation indices showed that genetic differentiation at all the three hierarchical levels was highly significant (*Fsc* = 0.13165, *Fst* = 0.71915 and *Fct* = 0.67657, *p* < 0.001).

### 2.2. Quantification of A. oxytropis and Swainsonine of Oxytropis Plants

Endophyte infection rates and swainsonine concentration in each individual plant were measured to see if these could be related to species. The levels of endophyte *A. oxytropis* ranged from 0.03 to 1.00 pg/ng as indicated by fungal/plant DNA ratios, and swainsonine contents ranged from 0.0006% to 1.19% as in plant dry mass (Table 2). *A. oxytropis* was detected in 9 out of the 17 species. These nine species all contained swainsonine. In these nine species, *O. glacialis* contained the highest level of swainsonine, but surprisingly, only one in five samples had detectable *A.* sect. *Undifilum*, and in that single sample, the levels were low. 

### 2.3. Interaction between Genetics and Environment and Their Integrated Effect on Endophyte Symbiosis and Swainsonine Production

To determine how host variation, endophyte interactions and environment could be related, a series of stepwise assessments were undertaken. The two genetic clades in the neighbour-joining tree of *Oxytropis* species did not distinguish the *A. oxytropis* positive or negative or the chemotypes of plants, e.g., the common ancestor of one clade did not correspond to monophyletic inheritance of symbiosis or toxicity (Figure 1, Table 2). Thus, the genetic variation of the host plants after speciation could be linked to the presence or absence of the endophytic fungi, such as genetic drift. We then analysed whether environmental factors play a role in plant genetic variation. To do this, we analysed how geographical and genetic distances of samples could be correlated. Correlation between geographical distance was weakly correlated with genetic distance (r = 0.2560, *p* < 0.001, *n* = 39) (Tables S4 and S5).

We further explored whether environmental factors had any impact on genetic diversity and the levels of endophytic fungi and swainsonine content in the *Oxytropis* plants (Table 3). Results showed that environmental variation had no influence on host genetic diversity in the populations (*n* = 39). The endophyte amount in the detectable individual samples was weakly correlated with annual average temperature (r = 0.301, *p* < 0.05, *n* = 59) and relative humidity (r = −0.322, *p* < 0.05, *n* = 59). The swainsonine content of each individual plant was weakly correlated with annual average water vapor pressure (r = −0.286, *p* < 0.05, *n* = 59) and moderately with relative humidity (r = −0.433, *p* < 0.01, *n* = 59), percentage sunlight (r = 0.363, *p* < 0.01, *n* = 59) and altitude (r = 0.341, *p* < 0.01, *n* = 59). 

**Figure 6:**
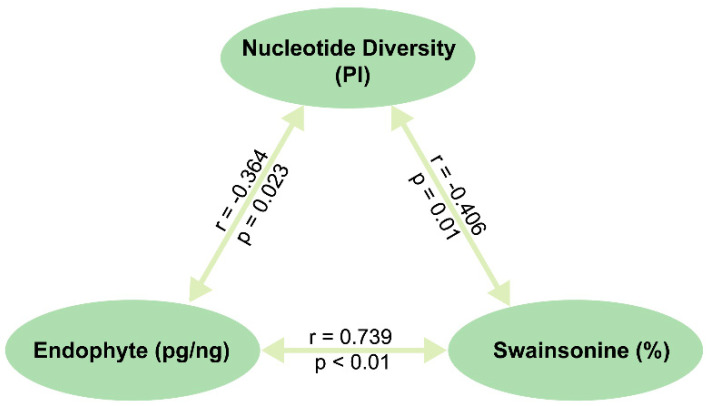
Correlation analysis results between nucleotide diversity (PI), concentration of *A. oxytropis* (pg/ng) and swainsonine (%) in the 39 *Oxytropis* populations.

We then assessed whether there was a correlation between three factors, namely genetic diversity (as indicated by nucleotide diversity), endophyte and swainsonine content in the 39 *Oxytropis* populations (Figure 6). Results showed that there was a weak negative correlation between genetic diversity and endophyte levels (r = −0.364, *p* < 0.05, *n* = 39). These results indicated that genetic diversity in plant populations may have affected the infection of endophytic fungi to a certain extent, resulting in the different levels of the endophytic fungi in plants. Secondly, there was a moderate negative correlation between plant genetic diversity and swainsonine content (r = −0.406, *p* = 0.01, *n* = 39). Finally, there was a considerable correlation between endophytic and swainsonine levels in populations (r = 0.739, *p* < 0.01, *n* = 39). 

## 3. Discussion

In this research, we examined the genetic variation of common *Oxytropis* spp. in China using genome-wide SNP data. Genetic differentiation was significant at the three genetic hierarchies amongst these species, but such differentiation was not influenced by environmental variation. Previous assessments of phylogenetic relationships and genetic diversity with *Oxytropis* plants employed the molecular approaches used at the time [29,30,31,32]. Most of these studies were based solely on such as 5.8SrDNA, ITS and cpDNA fragments (*trnH-psbA*, *trnL–trnF*, and *trnS–trnG*), or AFLP markers to reveal the developmental relationship between certain specific *Oxytropis* species [32,33,34,35,36,37]. Thus, the resulting inferences of *Oxytropis* evolutionary history and the division of certain morphologically similar species could be reappraised, especially with the development of high-throughput sequencing to identify SNPs. This approach has allowed the better characterisation of the determinants of plant–microbe interactions [38]. 

There are some obvious geographical barriers that could influence the distribution of *Oxytropis* species such as the Qinghai-Tibet Plateau and the Qilian and Helan mountain range. Such geographical effects were also seen in previous studies on *Oxytropis* campestris var. *chartacea* in eastern North America [32] and these may have caused the isolation of species and populations. Indeed, based on the phylogenetic relationships, most of the *Oxytropis* species we examined belong to their own clade showing diversity linked to speciation. However, it is worth noting that *O. giraldii* and *O. melanocalyx* could not be resolved. Given that these two species are morphologically alike and originated from a common ancestor, it may be that these could be regarded as subspecies but this awaits confirmation following further investigation. Alternatively, the hybridisation barrier may not function in these two species, so they are able to cross-pollinate and may have caused genetic introgression [39]. In addition, the traditional placement of *O. glacialis* to *Oxytropis* subgen. *Oxytropis* and *O. aciphylla* to *Oxytropis* subgen. *Tragacanthoxoytropis* may need to be revisited as both species are genetically close to the species in the *Oxytropis* subgen. *Orobia*. In a previous study, ITS and trnL-F sequences were used to group *O. glacialis* and other *Oxytropis* subgen. *Oxytropis* together [40], indicating that different molecular identification methods may have different results. Although PCA and admixture models resulted in six and eight clusters in the 17 species, they generally agreed with the phylogenetic tree in genetic relatedness. Genetic introgressions were observed in the admixture analysis. This may indicate those species diversified in specification in relatively recent evolutionary events.

In order to examine the endophyte levels, we developed a new qPCR primer set to avoid the nonspecific amplification observed in our previous work [27]. The new primer set was successfully used in the 17 species, demonstrating the effectiveness for future detection of the endophyte. There were significant differences in *A. oxytropis* content among different species of *Oxytropis*, but this was not detected in eight *Oxytropis* species. Swainsonine was detected in all of the 17 species, including species with traceable amount. This may be due to the highly sensitive swainsonine detection method we used in this study. According to a previous classification, four species—*O. glacialis*, *O. deflexa*, *O. sericopetala* and *O. falcata*—are type I *Oxytropis* plants, which indicates potent toxicity. Three species, previously reported as toxic locoweeds—*O. glabra*, *O. ochrocephala* and *O. kansuensis*—are less toxic due to having intermediate swainsonine content. According to previous reports, among the many *Oxytropis* species, nine of them—*O. ochrocephala*, *O. glabra*, *O. kansuensis*, *O. glacialis*, *O. falcata*, *O. sericopetala*, *O. deflexa*, *O. latibracteata* and *O. hirta*—are the most common poisonous *Oxytropis* species found in China [6,41,42]. However, our result suggested that *O. latibracteata* is only a Chemotype II plant, and thus much less toxic or considered as non-toxic. Chemotype I and Chemotype II plants were found in the same species in various locations (Table S4), which could suggest that conversion between toxic or non-toxic *Oxytropis* plants is possible. In *O. sericea*, the content of *A. oxytropis*, host plant site, biological stage, planting site and the genotype of the endophyte would all possibly affect the levels of swainsonine [18,19,23,43]. Therefore, we speculate that the results of the above differences may also be related to the joint action of the amount of endophyte, climate environment and soil microbes as well as other factors, which all need to be further studied.

In this study, endophyte levels and swainsonine concentrations were fairly well correlated in the 17 species surveyed. However, in some species, for example *O. glacialis*, endophyte infection rates and levels were low but this species had the highest swainsonine concentration. This may reflect the presence of other swainsonine-producing strains [20], such as either other *A. oxytropis*-related fungi in *A.* sect. *Undifilum* which were not able to be detected by the qPCR primer set, or other endophytes, as many fungi contain the *swnK* gene (as suggested by a blast search against the NCBI database, data not shown). It is also possible that swainsonine is produced in other organs in the host, but transferred into the leaf via vascular tissues. Similar results were found in our previous study [27]. 

Previously, we speculated that G × E interactions influence swainsonine biosynthesis in an *Oxytropis* species [27]. In this study, we took this further in different *Oxytropis* species at the genus level. Since *A. oxytropis* is considered to be vertically transmitted only, its presence in different *Oxytropis* species may indicate such symbiosis was shared in the common ancestor of the *Oxytropis* genus. During evolution, genetic divergence of the common ancestor of a certain lineage may result in the loss of *A. oxytropis* in different species, if an unknown selection force has worked as a driver [42,43,44]. If this were the case, the genetic similarity of several host species should reflect the presence or absence of *A. oxytropis* symbiosis. However, we observed that *Oxytropis* species with or without the endophyte symbiosis did not cluster together in separate lineages. On the other hand, we found that the genetic variation of the host is negatively correlated with the content of *A. oxytropis* and swainsonine at the population level. Therefore, we speculate that genetic drift could have led to the gradual loss or tune-down of this symbiotic capability. This could also indicate that the symbiosis may be selectively neutral as the hosts evolve and is being stochastically lost in several species. Meanwhile, several environmental factors were correlated with the amount of *A. oxytropis* and swainsonine. Since the levels of endophyte and swainsonine are positively correlated, environment may have an indirect or direct impact on swainsonine biosynthesis. Such a mechanism needs further investigation.

## 4. Conclusions

In this study we characterised the genetic diversity and levels of endophyte symbiosis and swainsonine in 17 common *Oxytropis* species in China. We showed that speciation, possibly with genetic drift, rather than the genetic lineage, is associated with the fixation or loss of endophyte and swainsonine production, which appears to be a random event. Similar to the influence at the species level, G × E also affects endophyte symbiosis and swainsonine production in the *Oxytropis* genus. Our study will provide better understanding about the evolutionary basis of *A. oxytropis* symbiosis and swainsonine biosynthesis in locoweeds.

## 5. Materials and Methods

### 5.1. Plant Materials and Genomic DNA Extraction

A total of 170 individuals from 17 *Oxytropis* species were collected from grasslands in China during the summer of 2017 (Figure 4 and Figure 5) and the associated geographical information was recorded (Table S3). Upon collection, foliar tissue was desiccated in airtight bags with silica. Voucher samples and specimens were stored in Northwest University, China. Taxonomic identification was performed by Prof. Chaoyang Chang from Northwest Agriculture and Forest University, who specialises in *Oxytropis* and Fabaceae. Leaves were ground to powder using a Tissue Lyser II bead-mill (Qiagen, Hilden, Germany). DNA was extracted from the ground tissue using the DNEasy plant mini-kit (Qiagen). DNA integrity was determined on 1% agarose gel and the concentration was measured on a NanoDrop ND-1000 Spectrophotometer (NanoDrop Technologies, Wilmington, DE, USA). DNA samples were aliquoted and stored at −20 °C and −80 °C until use.

### 5.2. SLAF Sequencing and SNP Calling

Genotypes of 170 individuals from 17 species of *Oxytropis* were analysed by SLAF-seq, a genotyping by sequencing approach. SLAF-seq library construction and high-throughput sequencing were performed as described previously [45]. After a quality check of the sequencing data, the BLAT program [46] was used to cluster SLAF tags for each sample according to sequence similarity to obtain polymorphic SLAF tags. Then, the “mem” program of BWA was used to compare the filtered high-quality data to the reference genome of *O. ochrocephala* available in our laboratory (PRJNA771236 in NCBI), and the comparison parameters were set as default. The comparison result was outputted in sam format. The “SortSam” command of Picard 1.107 was used to sort the sam files. Next, GATK (Genomics Analysis K V3.8) [47] was used to detect SNPs, which were further filtered to obtain high-quality SNP markers. The filter conditions were set as follows: (1) Coverage multiple ≥ 16, major SNP coverage multiple ≥ 8, major SNP coverage multiple /minor SNP coverage multiple ≥ 5; (2) MQ (RMS Mapping Quality) ≥ 25; (3) QD (Variant Confidence/Quality by Depth) ≥ 2.

### 5.3. Characterisation of Genetic Diversity and Genetic Structure

To reveal the genetic structure among all individuals, we constructed the phylogenetic tree and performed principal component analysis (PCA), and inferred a group genetic structure. Using the neighbor-joining algorithm, the Kimura 2-parameter model was selected to construct the phylogenetic tree of 17 *Oxytropis* species with 1000 bootstraps using MEGA7.0 [48]. *Medicago truncatula* (Mt) was selected as the outgroup and used as the root. The population structure was analysed using ADMIXTURE [49], and the cluster number (K value) of all samples was determined for K = 1–10. The optimal cluster number K (best taxa) was determined as the one with the minimum cross-validation error rate (CV). GCTA [50] (http://www.complextraitgenomics.com/software/gcta/ (accessed on 30 November 2021)) was used for the PCA analysis.

In order to analyse the genetic structure of *Oxytropis*, in addition to calculating genetic distance, AMOVA [51] was used to estimate the degree of genetic differentiation among and within populations/species.

### 5.4. Determination of Endophyte and Swainsonine Concentration

Most of the samples used for SLAF-seq were assessed for levels of *A. oxytropis*. A quantitative real-time PCR method (qPCR) was used to determine the amount of the endophytic DNA of *A. oxytropis* which was contained in the extracted total genomic DNA from the plant [52]. In view of the limitation of the previous method for quantifying endophyte *A. oxytropis* in two *Oxytropis* species due to unspecific amplification (in *O. kansuensis* and *O. ochrocephala*) [27], a new primer set was developed based on a polyketide gene *swnK*. A standard curve (Figure S3) was established using a dilution series of the endophyte DNA extracted from pure fungal culture, and the amount of endophyte calculated. All the qPCR reactions were performed in quadruplicate using the forward and reverse primer SwnK-q-S/SwnK-q-AS (Table S6). The reaction conditions are described in Table S7 and in Figure S4.

Swainsonine extraction was performed as described by [53]. The samples were analysed by flow infusion electro-spray ionisation high-resolution mass spectrometry (FI-ESI-MS). The mass spectra were acquired on an Accela (ThermoFinnigan, San Jose, CA, USA) ultra-performance liquid chromatography system coupled to an Exactive Orbitrap (ThermoFinnigan, San Jose, CA, USA) mass spectrometer. For determination, 20 μL of sample was injected and delivered to the ESI source via ultra-pure H_2_O (18.2 Ω) and pre-mixed HPLC grade MeOH (Fisher Scientific, Hampton, NH, United States) or a flow solvent (mobile phase) at a ratio of 3:7. For the first 1.5 min, the flow rate was 200 μL·min^−1^ and 600 μL·min^−1^ for the remaining 1.5 min of the method. Both ionisation modes were acquired at the same time. The maximum injection time was 205 ms and the mass resolution was 100,000 with an automatic gain control (AGC) of 5 × 105 for both modes of ionisation. Swainsonine peak intensities (*m/z* 174.1124 [M+H]^+^) were converted to ug/mL concentration based on the derivation of a standard curve using commercially obtained swainsonine (Santa Cruz Biotechnology, Dallas, TX, USA) that was assessed by FI-FI-ESI-MS.

Endophyte levels were calculated and represented as the DNA content of endophyte *A. oxytropis* in plant DNA content (pg/ng). Swainsonine content in each sample was obtained and represented as the percentage of swainsonine in plant dry weight (%). To keep consistent with previous studies, we applied a 0.0001% limit for swainsonine. Any value below this value was considered as not detected [50].

### 5.5. Statistical Analysis

In order to obtain more accurate plant genetic distance and geographic distance analysis results, after re-dividing all samples into 39 populations according to sampling locations, we used the K2P model in MEGA 7.0 to recalculate the genetic distance matrix. The geographic distance matrix between any two sampling locations was calculated by the latitude and longitude information in Google Earth (V7. 15. 1557). Finally, the “ade4” program in the R package was used to conduct Mental tests to analyse the correlation between the genetic distance and geographical distance of *Oxytropis* spp.

We also examined the influence of different climate factors and altitude on plant genetic diversity, endophytic fungus content and swainsonine content. Climate data at county level relevant to the 39 populations for 1988–2018 were downloaded from the China Meteorological Data Network (http://data.cma.cn/ (accessed on 20 September 2019)), and Kriging interpolation on the data was performed in ArcGIS 10.2 software based on the coordination of the 39 populations (Table S2). To analyse the correlation for endophyte and swainsonine, only individuals with detectable endophyte were used for Pearson correlation analysis by SPSS Statistics v22.

Finally, in order to show the relationship between the genetic diversity and the contents of *A. oxytropis* and swainsonine in *Oxytropis*, we first characterised the genetic diversity parameter nucleotide diversity (PI) in the 39 populations using vcftools [54]. Then we used SPSS Statistics V22 to perform a Spearman bivariate correlation analysis of nucleotide diversity, endophytic fungus content and swainsonine.

## Figures and Tables

**Table 1:** Analysis of molecular variance (AMOVA) of the genetic diversity in *Oxytropis*.

Source of Variation	d.f.	Sum of Squares	Variance Components	Percentage of Variation	Fixation Indices
Among 17 species	16	30,048.556	175.96679 Va	67.66	*Fsc* = 0.13165 ***
Among populations within each species	22	2388.915	11.07451 Vb	4.26	*Fst* = 0.71915 ***
Among individuals within each of the 39 populations	131	9569.124	73.04675 Vc	28.09	*Fct* = 0.67657 ***
Total	169	42,006.594	260.08805	100	

Asterisks (***) indicate significant genetic differentiation (*p* < 0.001).

**Table 2:** Endophytic fungi and swainsonine content in *Oxytropis*.

Species	Number of Samples	Number of Endophyte + Samples	Endophyte Concn. (Mean pg/ng ± S.E.)	Swainsonine (Mean % ± S.E.)	Chemotype
*O. glacialis*	5	1	0.03 ± 0.03	1.1900 ± 0.3278	Chemotype I
*O. deflexa*	9	9	0.99 ± 0.14	0.3267 ± 0.0788	Chemotype I
*O. sericopetala*	9	9	1.00 ± 0.05	0.2140 ± 0.0445	Chemotype I
*O. falcata*	15	13	0.60 ± 0.08	0.1643 ± 0.0423	Chemotype I
*O. glabra*	10	5	0.51 ± 0.17	0.0668 ± 0.0164	Intermediate
*O. ochrocephala*	13	9	0.26 ± 0.09	0.0479 ± 0.0177	Intermediate
*O. kansuensis*	22	6	0.06 ± 0.02	0.0145 ± 0.0042	Intermediate
*O. giraldii*	14	4	0.22 ± 0.11	0.0030 ± 0.0019	Chemotype II
*O. ochrantha*	5	3	0.10 ± 0.04	0.0022 ± 0.0012	Chemotype II
*O. latibracteata*	10	n.d.	n.d.	0.0022 ± 0.0008	Chemotype II
*O. melanocalyx*	10	n.d.	n.d.	0.0021 ± 0.0014	Chemotype II
*O. myriophylla*	10	n.d.	n.d.	0.0015 ± 0.0007	Chemotype II
*O. merkensis*	4	n.d.	n.d.	0.0010 ± 0.0005	Chemotype II
*O. psamocharis*	10	n.d.	n.d.	0.0008 ± 0.0001	Chemotype II
*O. bicolor*	6	n.d.	n.d.	0.0007 ± 0.0001	Chemotype II
*O. ramosissima*	10	n.d.	n.d.	0.0007 ± 0.0001	Chemotype II
*O. aciphylla*	8	n.d.	n.d.	0.0006 ± 0.0001	Chemotype II

Swainsonine contents of >0.1% or <0.01% are considered as the thresholds to define the plants as chemotype I or chemotype II plant [21], based on which *Oxytropis* appeared to group into three categories. Four species (*O. glacialis*, *O. deflexa*, *O. sericopetala* and *O. falcata*) contained swainsonine contents of >0.1%, thus were Chemotype I. *O. glabra*, *O. ochrocephala* and *O. kansuensis* were intermediate between Chemotypes I and II; and all the other 10 species were Chemotype II plants.

**Table 3:** Correlation analysis between environmental factors, altitude and content of endophytic fungi and swainsonine.

	Nucleotide Diversity (PI)	Endophyte (pg/ng)	Swainsonine (%)
Annual average precipitation (mm)	−0.062	−0.090	−0.077
Annual average air pressure (hpa)	−0.004	−0.004	−0.196
Annual average wind speed (m/s)	0.037	−0.004	0.063
Annual average temperature (°C)	−0.146	0.301 *	−0.038
Annual average water vapor pressure (hpa)	−0.115	0.072	−0.286 *
Annual relative humidity (%)	−0.012	−0.322 *	−0.433 **
Annual sunlight percentage (%)	0.087	0.213	0.363 **
Altitude (m)	0.036	−0.060	0.341 **

Note: * *p* < 0.05; ** *p* < 0.01.

## Data Availability

The data presented in this study are available on request from the corresponding author.

## Supplementary Materials

The following supporting information can be downloaded at: https://www.mdpi.com/article/10.3390/toxins14030181/s1, Figure S1: CV value of ADMIXTURE, Figure S2: Results of ADMIXTURE, Figure S3: A standard curve of qPCR reaction, Figure S4: Reaction procedures of qPCR reaction, Table S1: Genetic distances separating the 17 *Oxytropis* species, Table S2: The climate factors, altitude, nucleotide diversity, endophytic fungi and swainsonine content information of 39 populations of *Oxytropis* species, Table S3: The sampling information of *Oxytropis* species, Table S4: Genetic distance of Kimura 2-parameter matrix of *Oxytropis* species, Table S5: Geographic distance of Kimura 2-parameter matrix of *Oxytropis* species, Table S6: Gene primer information, Table S7: qPCR reaction system.
