# Supplementary material for: Host-Species Variation and Environment Influence Endophyte Symbiosis and Mycotoxin Levels in Chinese Oxytropis Species
Source: Toxins (Basel). 2022 Feb 28;14(3):181. doi: 10.3390/toxins14030181 (PMC8948792; doi:10.3390/toxins14030181)
Supplement: Supplementary file 1 [file toxins-14-00181-s001.zip › 1530149-supplementary/supplementary/supplementary.pdf]

toxins

MDPI

Supplementary Materials: Host-Species Variation and Environment Influence Endophyte Symbiosis and Mycotoxin Levels in Chinese *Oxytropis* Species

Chenchen Guo, Li Zhang, Qianqian Zhao, Manfred Beckmann, Helen Phillips, Huizhen Meng, Chonghui Mo, Luis A. J. Mur and Wei He

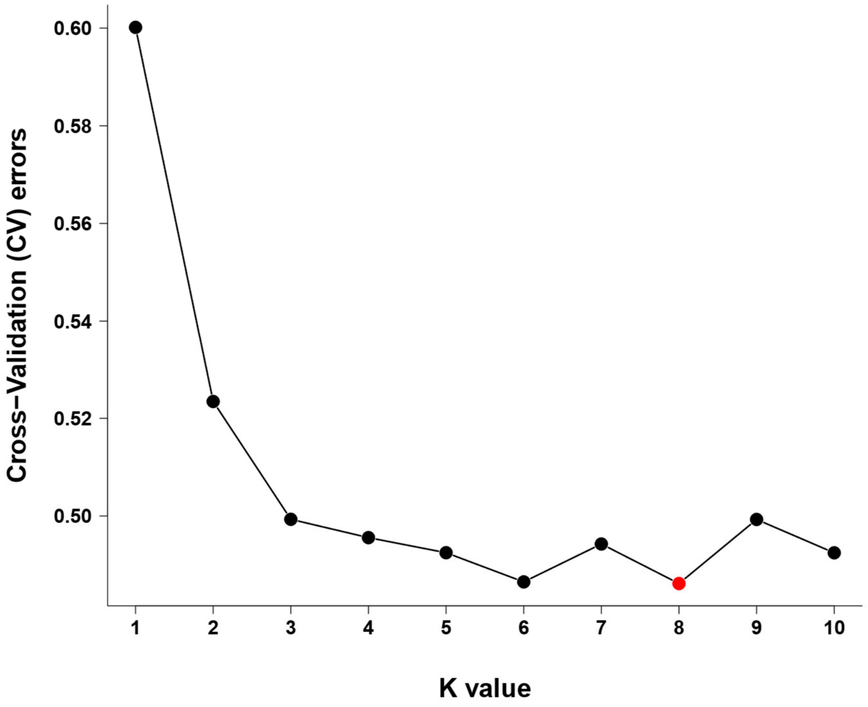

Figure S1. CV value of admixture. The first significant decrease in ΔK indicates the best explanatory K=8, suggesting eight genetically distinct populations.

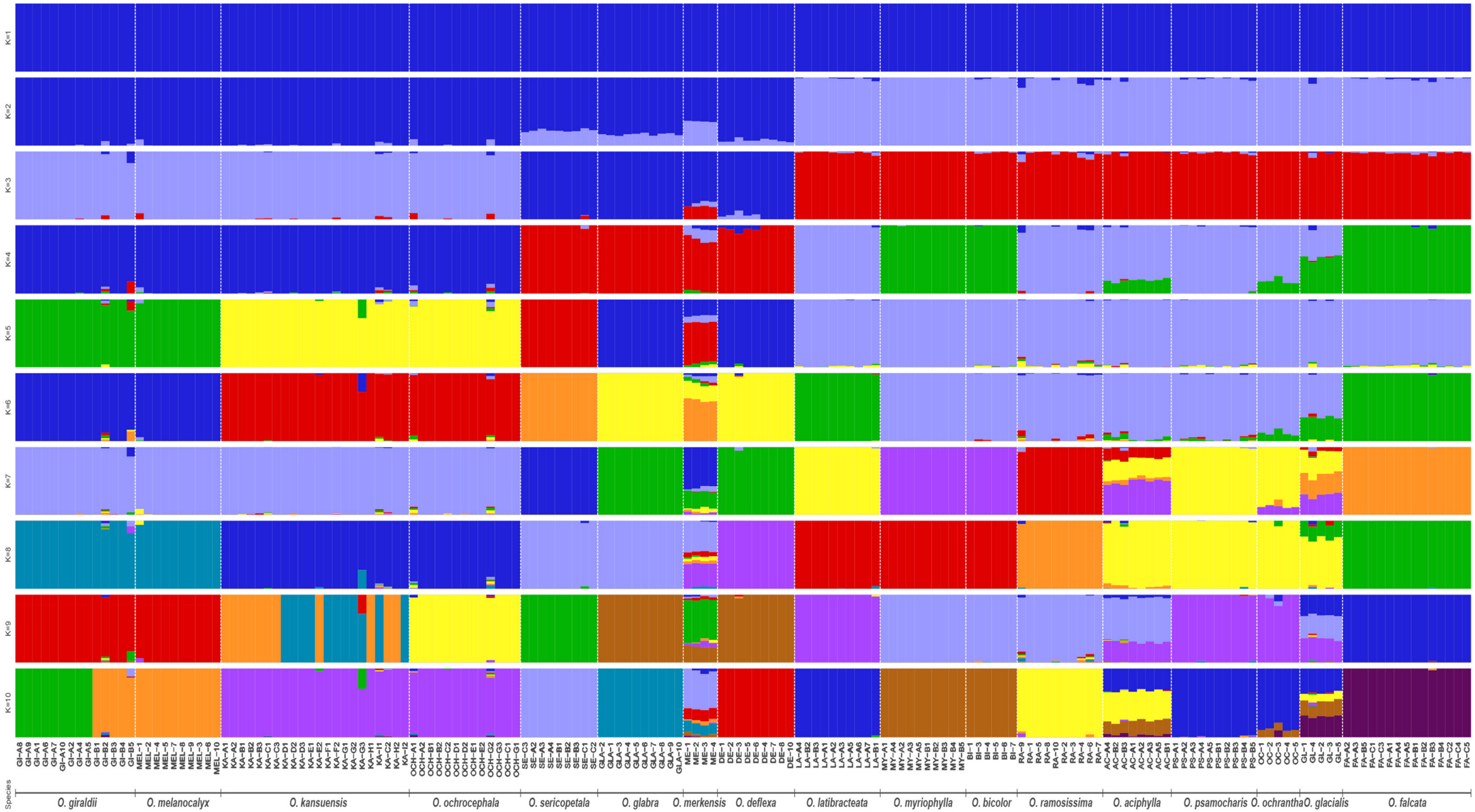

Figure S2. Results of admixture.

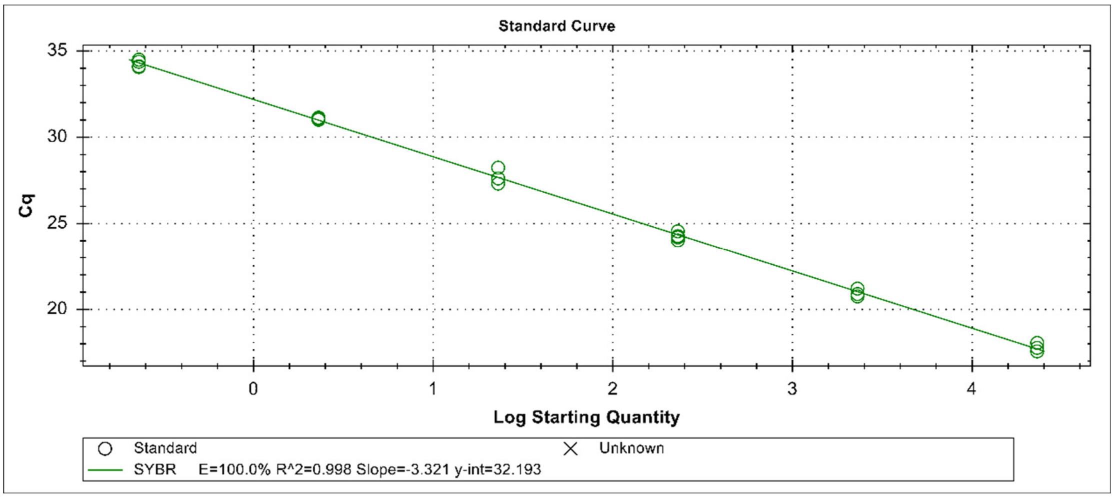

Figure S3. A standard curve of qPCR reaction. y = - 3.321x +32.193, R^2=0.998, E=100.0%, the range of Cq value is 17.57-34.50. Indicating that there is a good linear relationship within the range of the standard curve dilution mass concentration, and the reaction amplification effect is ideal, and the amplification system is stable.

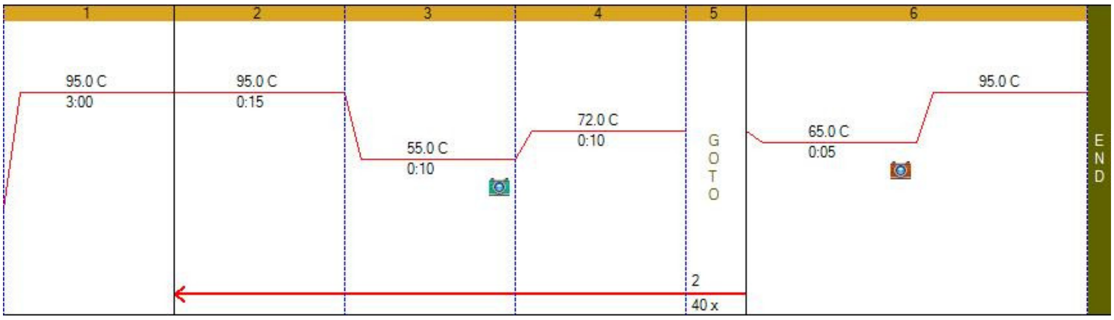

Figure S4. Reaction procedurse of qPCR reaction.
